# Circular RNAs in Hepatocellular Carcinoma: Emerging Functions to Clinical Significances

**DOI:** 10.3389/fonc.2021.667428

**Published:** 2021-05-14

**Authors:** Yucheng Zhang, Yali Wang

**Affiliations:** ^1^ Scientific Research Center, China–Japan Union Hospital of Jilin University, Changchun, China; ^2^ Department of Blood Transfusion, China–Japan Union Hospital of Jilin University, Changchun, China

**Keywords:** prognosis, diagnosis, biomarker, circular RNA, hepatocellular carcinoma

## Abstract

Hepatocellular carcinoma (HCC) is the most common primary cancer of the liver and carries high morbidity and mortality. Diagnosing HCC at an early stage is challenging. Therefore, finding new, highly sensitive and specific diagnostic biomarkers for the diagnosis and prognosis of HCC patients is extremely important. Circular RNAs (circRNAs) are a class of non-coding RNAs with covalently closed loop structures. They are characterized by remarkable stability, long half-life, abundance and evolutionary conservation. Recent studies have shown that many circRNAs are expressed aberrantly in HCC tissues and have important regulatory roles during the development and progression of HCC. Hence, circRNAs are promising biomarkers for the diagnosis and prognosis of HCC. This review: (i) summarizes the biogenesis, categories, and functions of circRNAs; (ii) focuses on current progress of dysregulated expression of circRNAs in HCC with regard to regulation of the tumor hallmarks, “stemness” of cancer cells, and immunotherapy; (iii) highlights circRNAs as potential biomarkers and therapeutic targets for HCC; and (iv) discusses some of the challenges, questions and future perspectives of circRNAs research in HCC.

## Introduction

Hepatocellular carcinoma (HCC) is the fourth-leading cause of cancer-related death overall worldwide, and the fastest-growing cause of cancer-related deaths in the United States (1, 2). The major risk factors for HCC include excessive consumption of alcohol, nonalcoholic fatty liver disease, diabetes mellitus, infection by the hepatitis B virus and/or hepatitis C virus, and dietary exposure to aflatoxins (3). Although surgery is first-line treatment, only 5–10% of HCC patients are eligible for surgery because HCC is frequently diagnosed at a late stage, and its prognosis is poor. Therefore, the most urgent needs are to elucidate the molecular mechanisms underlying the development and progression of HCC, and to find sensitive markers for the diagnosis and prognosis of HCC (4, 5).

Accumulating evidence has demonstrated that some circular RNAs (circRNAs) are involved in HCC carcinogenesis and progression, including proliferation, angiogenesis, apoptosis, invasion and migration. circRNAs are a novel type of non-coding (nc)RNAs and harbor a covalently closed loop structure with neither 5′ to 3′ polarity nor a polyadenylated tail (6). Therefore, circRNAs are featured by the properties of remarkable stability, long half-life, resistance to exonucleolytic RNA decay, and evolutionary conservation (7, 8). Furthermore, studies on circRNAs have exhibited that they are expressed in given cell type-, tissue-, developmental stage- and disease-specific patterns (9–11).

Initially, circRNAs were hypothesized to be byproducts generated by aberrant splicing events. In recent years, thanks to the rapid development of high-throughput sequencing techniques and bioinformatics methods, many circRNAs have been identified in various human tissues and shown to be involved in diverse biological and pathological processes of various tumor types (12–15). Of note, specific deregulated circRNAs have been shown to participate in the biological and pathological processes of HCC, indicating that these dysregulated circRNAs may become potential biomarkers and therapeutic targets for HCC (16–19).

In this review, we summarize the biogenesis, types, and biological functions of circRNAs. In particular, we highlight the biological roles of circRNAs in HCC and the potential clinical value of circRNAs as novel biomarkers and therapeutic targets for HCC. We also discuss the main problems and perspectives for the utility of circRNAs in HCC.

## Biogenesis and Types of CIRCRNAs

Although the exact mechanisms of circRNAs biogenesis have not been fully elucidated, emerging studies have revealed that circRNAs are generated mainly through noncanonical splicing of precursor messenger RNAs (pre-mRNAs) termed “backsplicing” (20). Different from the canonical splicing of pre-RNAs terminated with a 5′ cap and 3′ polyadenylated tails, circRNAs are characterized by their single-strand closed-loop structure, which is generated through the ligation of a downstream 5′ splice donor site and an upstream 3′ splice acceptor site (21–23).

**Figure 1:**
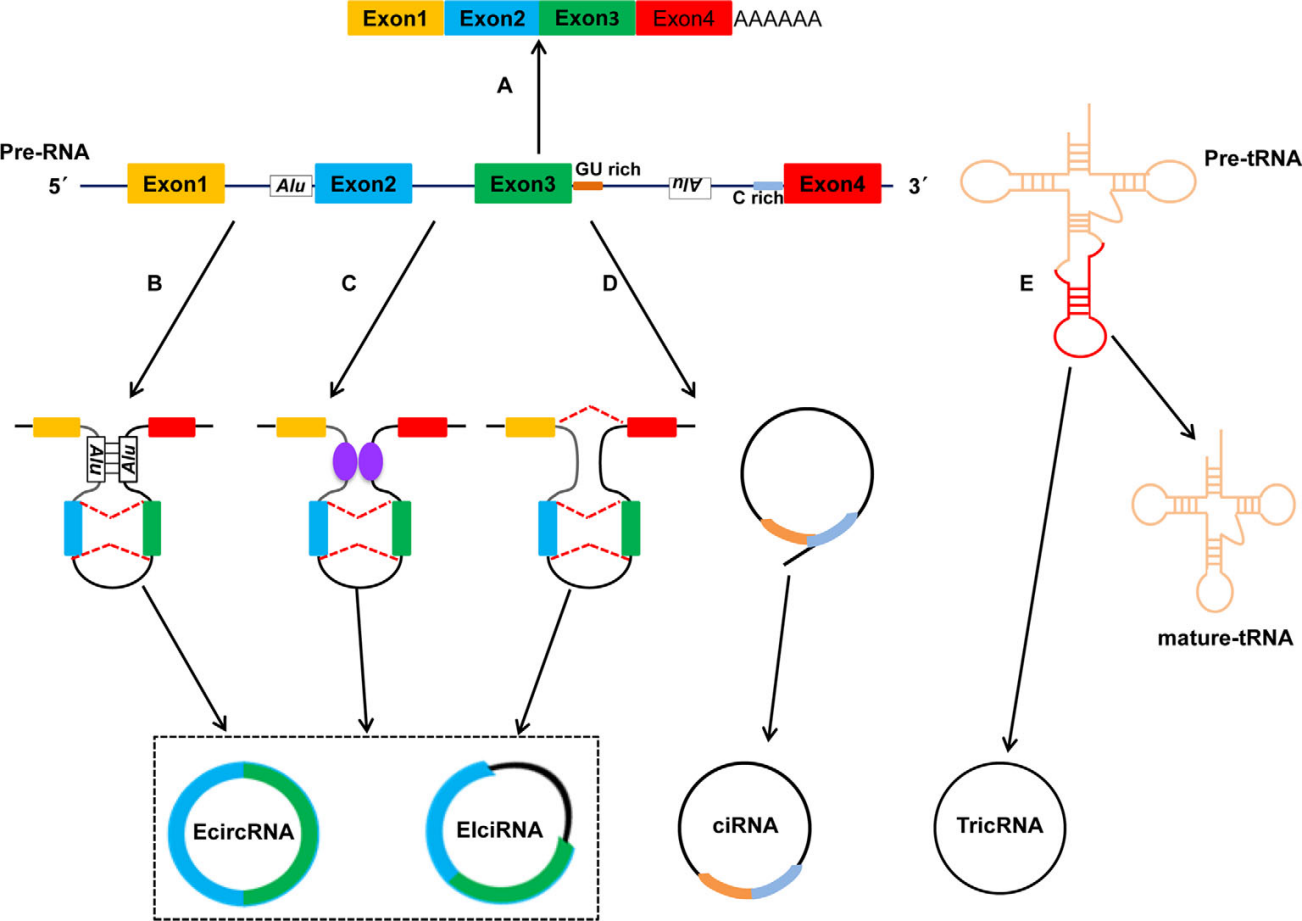
Biogenesis and types of circRNAs. Studies have identified four categories of circRNAs: EcircRNAs, EIciRNAs, ciRNAs and TricRNAs. The four types of circRNAs are formed by different mechanisms. **(A)** Canonical pre-mRNA splicing to produce a mature mRNA. **(B)** Intron pairing-driven circularization. **(C)** RBPs-associated circularization. **(D)** Lariat-driven circularization. **(E)** TricRNA is generated during pre-tRNA splicing.

Based on their origin of genomic regions, circRNAs are divided mainly into four types (**Figure 1**) (5, 24): exonic circRNAs (ecircRNAs), retained-intron or exon-intron circRNAs (EIciRNAs), intronic circRNAs (ciRNAs) and tRNA intronic circRNAs (tricRNAs). The vast majority of circRNAs is ecircRNAs, which account for >80% of identified circRNAs, and ecircRNAs are distributed predominantly in the cytoplasm (25, 26). Several studies have suggested that ecircRNAs have important roles in regulation of gene expression because they sponge microRNAs (miRNAs) and/or interact with RNA-binding proteins (RBPs) (27, 28). Unlike ecircRNAs, EIciRNAs and ciRNAs represent only a small fraction of circRNAs, and they are located mainly in nucleases and can regulate expression of their parental mRNAs (20).

Up to now, three hypothetical models of the mechanisms of circRNAs biogenesis have been proposed (21, 29): lariat-driven circularization (also called “exon skipping”), intron pairing-driven circularization, and RBPs-mediated circularization. In the lariat-driven circularization model, a large lariat containing one or more skipped exon(s) is produced. Then, the lariat may undergo backsplicing and the intronic sequences in the lariat are removed, ultimately resulting in the generation of ecircRNAs or EIciRNAs (30, 31). With regard to the intron pairing-driven circularization model, the pairing structure of circRNAs can be accomplished by the direct base-pairing of the introns flanking either inverted repeats (e.g., Alu elements) or complementary sequences (32, 33). In terms of the RBPs-mediated circularization model, RBPs or *trans*-factors play an important part in circRNAs biogenesis because they facilitate or inhibit intron pairing (34); quaking (QKI) and muscleblind (MBL) proteins can bind to specific sequence sites within flanking introns. Subsequently, they link two flanking introns together, thereby promoting circularization and facilitating circRNAs biogenesis (35–37); Conversely, adenosine deaminases acting on RNA 1 (ADAR1) can inhibit circRNAs biogenesis by binding to double-stranded RNA and destabilizing RNA pairing (38, 39). Furthermore, Pagliarini and colleagues suggested that Sam68 binds Alus-rich introns in Survival of Motor Neuron (SMN) pre-mRNAs and promotes pre-mRNAs circularization, which shows that Sam68 may cooperate with inverted repeat Alus (IR-Alu) to favor circRNAs biogenesis (40). Noto et al. reported a conversed and novel model of RNAs circularization in which tRNA introns are spliced to form tricRNAs during splicing of pre-tRNA (41).

## Functions of CIRCRNAs

**Figure 2:**
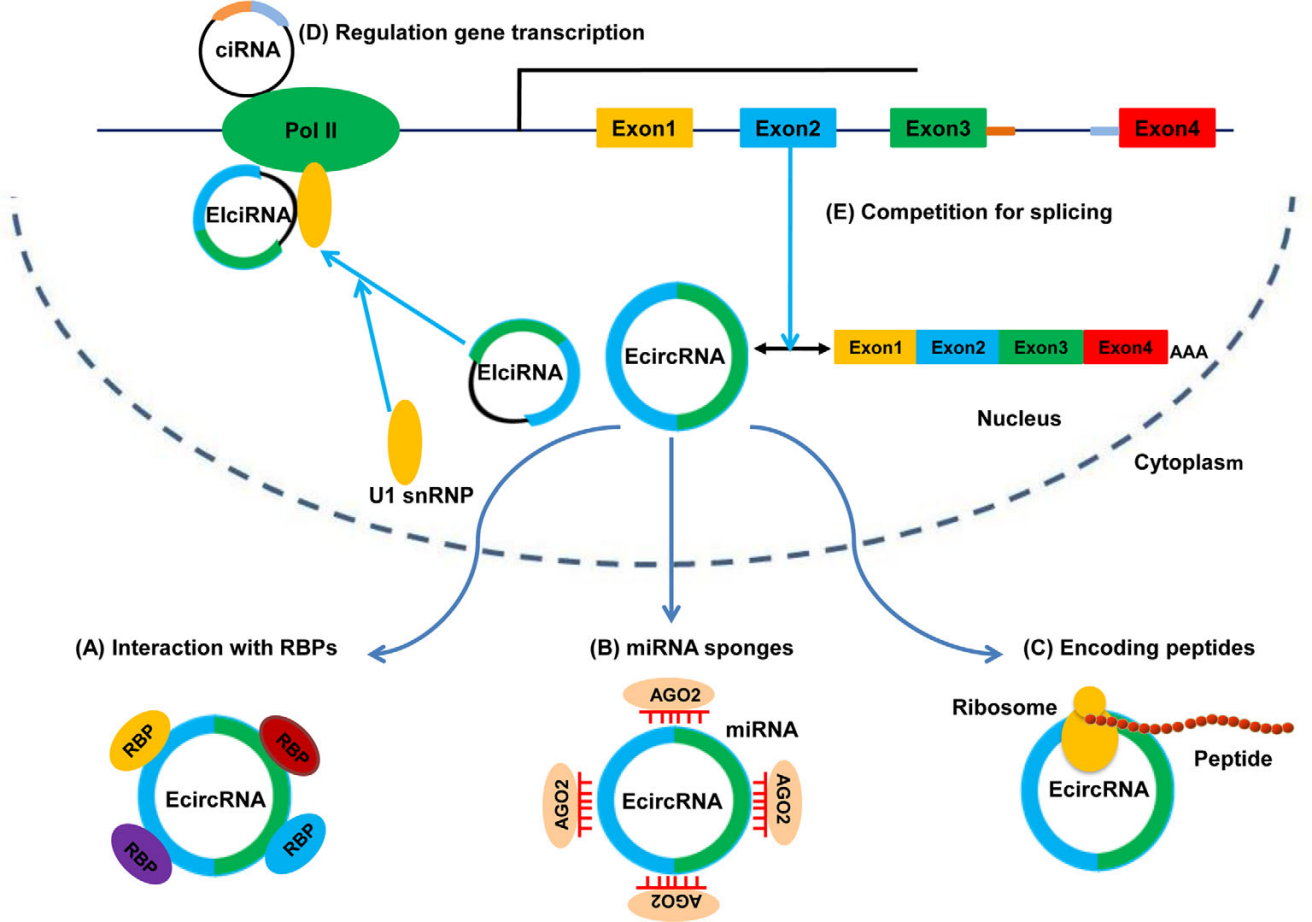
Functions of circRNAs. **(A)** Binding to RBPs. **(B)** Sponging miRNAs. **(C)** Translating into peptides or proteins. **(D)** Regulating gene transcription. **(E)** Competing with canonical splicing.

The functions of circRNAs have been investigated extensively. Several studies have revealed five major functions of circRNAs: binding to RBPs and serving protein scaffolds; sponging miRNAs to regulate target genes; encoding peptides or proteins; promoting transcription of parental genes; regulating alternative splicing. The five potential functions of circRNAs are shown in **Figure 2**.

### miRNA Sponges

The hallmark function of circRNAs is their ability to act as miRNA sponges to regulate gene expression. Most mechanistic studies on circRNAs have focused on miRNA sponges. miRNAs are a class of short ncRNAs. They can interact with mRNAs and induce post-transcriptional repression of target genes (42). Emerging evidence suggests that specific circRNAs contain multiple sites for miRNA binding, which indicates that circRNAs might act as endogenous RNAs (ceRNAs) or miRNA sponges to counteract miRNA-mediated gene degradation (43). A typical example of a miRNA sponge is ciRS-7, which is also termed as “cerebellum degeneration-related protein antisense” (CDR1). ciRS-7 contains >70 conserved miR-7 binding sites and binds miR-7 without being degraded, thereby increasing expression of miR-7-targeted mRNAs (11, 27). Studies have shown that the ciRS-7/miR-7 system may have important functions in the developing brain, tumorigenesis, and tumor progression (44, 45). Another example is sex-determining region Y (Sry) circRNA, which is derived from the testes of adult mice; Sry circRNA harbors 16 putative binding sites for miR-138 (27). Moreover, some circRNAs can act as miRNA sponges to different types of miRNAs even though these circRNAs lack multiple binding sites for a specific miRNA. For example, circ-ITCH acts as a tumor suppressor in multiple tumors, and inhibits progression of multiple tumor types by sponging different miRNAs (miR-17, miR-224, miR-22 and miR-214) and regulating the target genes of miRNAs (46–49). Similarly, circHIPK3 functions as an oncogene by sponging different miRNAs (including miR-7, miR-124, miR-558 and miR-637) involving multiple cancer types (e.g., colorectal, lung, and bladder) (50–54). Taken together, these findings indicate that some circRNAs act as sponges for miRNAs, and that the circRNA/miRNA interaction might be associated with human disease, such as cancer.

### Binding to Proteins or Serving as Protein Scaffolds

Studies have demonstrated that specific circRNAs act through interactions with proteins. One example comes from research on circMBL by Ashwal-Fluss and colleagues in 2014 (36). circMBL itself contained high MBL binding motifs, and there was a direct interaction and feedback loop between these two molecules. Abundant MBL protein promoted circMBL production by stimulating exon circularization. If MBL protein was reduced, it caused a significant decrease in circMBL production. Moreover, circPABPN1 and Poly(A)-binding protein nuclear 1 (PABPN1) mRNA competed for binding to HuR protein (a translational activator); massive binding of circPABPN1 to HuR inhibited HuR binding to PABPN1 mRNA, which caused a reduction in expression of PABPN1 mRNA (55). Besides, specific circRNAs can act as protein scaffolds to facilitate proteins assembly. For example, circFoxo3 serves as a protein scaffold to combine with CDK2 and p21, and the circFoxo3/CDK2/p21 ternary complex blocks cell cycle progression in response to cell overgrowth (56).

### Encoding Peptides or Proteins

Although circRNAs are considered to be ncRNAs and lack a 5′ cap or polyadenylated (A) tails, specific circRNAs can potentially encode peptides or proteins. In 2017, Legnini and colleagues were the first to report that circ-ZNF609 can be translated into a protein in a splicing-dependent and cap-independent manner in myogenesis, and provided a typical example of protein-coding circRNAs in eukaryotes (57). Moreover, studies have indicated that circRNA-encoded proteins (but not circRNAs themselves) have tumor-suppressive roles or tumor-promoting roles in cancer. For example, circPPP1R12A, which is produced by exons 24/25 of *PPP1R12A*, encodes a 73 amino-acid small peptide (named “PPP1R12A-73aa peptide”); it is circPPP1R12A-73aa rather than circPPP1R12A that has a tumor-suppressor role in colon cancer because it activates the Hippo-YAP signaling pathway (58). Similarly, circAKT3 encodes a 174aa protein (AKT3-174aa); AKT3-174aa (but not circ-AKT3) exerts a tumor-suppressive role by negatively regulating the phosphoinositide 3-kinase/protein kinase B (PI3K/Akt) signaling pathway in glioblastoma (GBM) (59). Overall, circRNAs may represent a novel type of peptide-coding RNAs, and circRNA-coding peptides also may have important potential clinical uses.

### Regulating Gene Transcription and Alternative Splicing

EIciRNAs and ciRNAs are found primarily in the nucleus, where they regulate gene transcription. For example, EIciRNAs (circEIF3J and circPAIP2) promote expression of their parental genes in a *cis*-acting manner. Mechanistically, an EIciRNA-U1 snRNP complex, which is formed *via* specific RNA-RNA interactions between EIciRNA and U1 snRNA, interacts further with Pol II at the promoters of parental genes to enhance transcription. Likewise, some ciRNAs (ci-ankrd52 and ci-sirt7), which are also retained in the nucleus and localized at the start sites of their parental-gene transcription, might function as positive regulators of Pol II and enhance the transcription of their parental genes (60).

circRNAs can also regulate alternative splicing. Backsplicing and linear pre-mRNA splicing share the same exons, so circRNAs circularization and mRNA splicing may compete with each other (36, 61). Therefore, the more an exon is circularized, the less the exon will be involved in the production of linearly processed mRNA (62, 63). As described above, circMBL competes with MBL pre-mRNA splicing and has a negative effect on linear splicing. Furthermore, some ecircRNAs may regulate the production of the canonical protein by sequestering mRNA start sites (31, 64).

## Roles of CIRCRNAS in HCC

Multiple lines of evidence indicate that some circRNAs are expressed aberrantly in HCC and have a vital regulatory role in the carcinogenesis and development of HCC. These dysregulated circRNAs can regulate various hallmarks of HCC, such as the cell cycle, angiogenesis, apoptosis, invasion and metastasis, and metabolism. Furthemore, some aberrantly expressed circRNAs are involved in regulating the “stemness” of cancer cells and immunotherapy of HCC through different mechanisms.

### circRNAs Regulate the Tumor Hallmarks in HCC

#### Cell Cycle Regulation by circRNAs in HCC

Dysregulation of the cell cycle is a main feature of malignancies. Cell cycle progression is regulated by coordinated regulators such as cyclins, cyclin-dependent kinases (CDKs), p53, c-Myc, and CDK inhibitor proteins (CKIs). The cyclin D/CDK4/6 complex is the central regulator of the transition of the G1 phase to the S phase of the cell cycle. Mitogenic signals activate cyclin D/CDK4/6 by phosphorylating retinoblastoma (RB) tumor-suppressor protein, which inhibits the activity of E2F transcription factors, and drives the expression of E2F target genes and promotes cell cycle progression (65, 66). The cyclin E/CDK2 complex has a critical role in the G1 phase, and is required for the progression from the G1 phase to the S phase, whereas cyclin A and cyclin B together with CDK1 are required for the progression from the G2 phase to the M phase (67). Cell cycle progression is also controlled by the activity of CKIs, which belong to the INK4 family (p15, p18 and p19) or Cip/Kip family (p21, p27 and p57). Furthermore, p53 and c-myc also have critical roles in the control of cell cycle progression.

**Figure 3:**
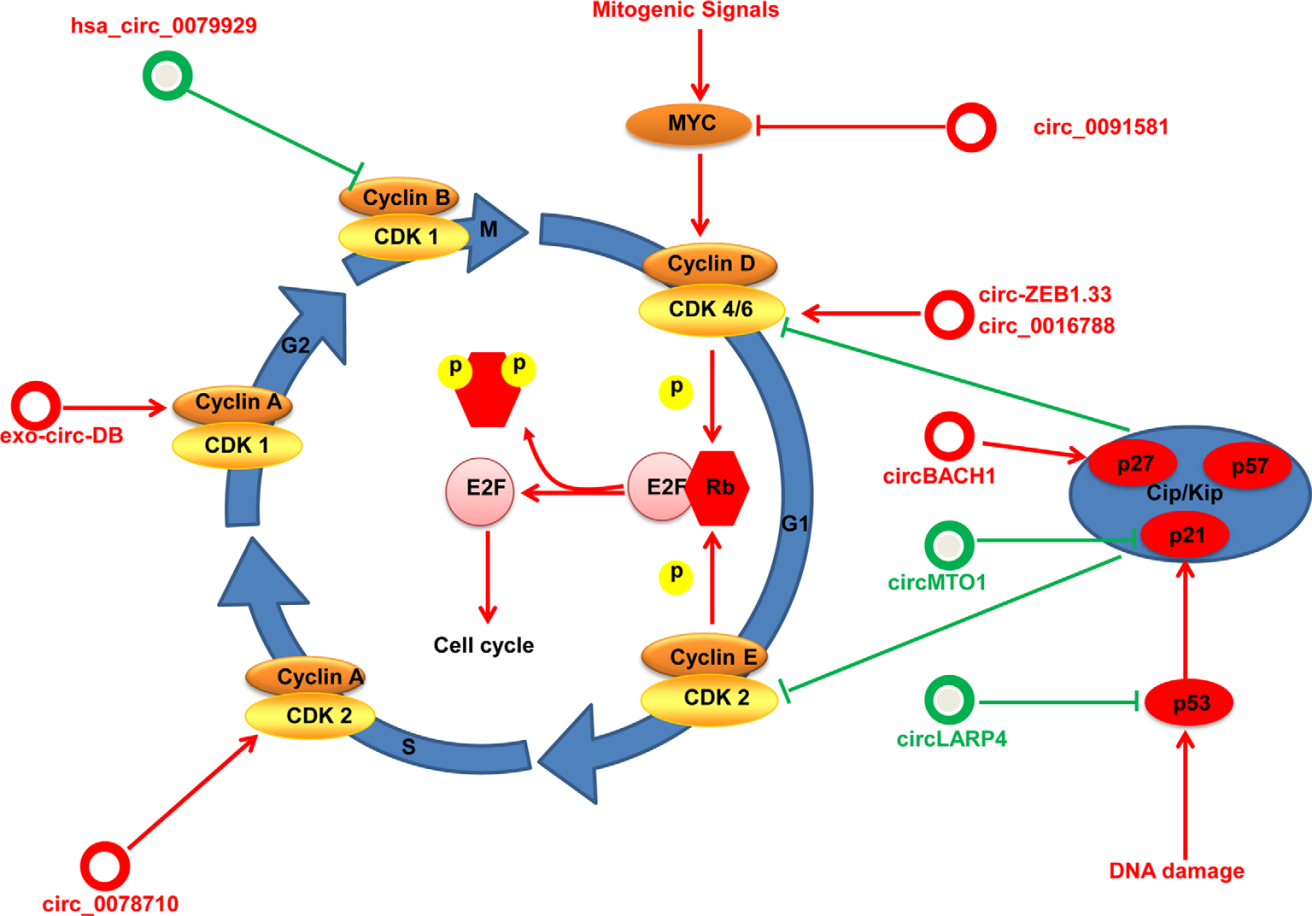
An overview to control of the cell cycle by circRNAs in HCC. The cell cycle consists of four phases: G1, S, G2, and M. circRNAs regulate the key regulatory molecules in HCC (cyclins, CDKs, CKIs, Myc and p53) by different mechanisms. circRNAs with proliferative potential are denoted as red circles whereas antiproliferative circRNAs are denoted as green circles.

Specific dysregulated circRNAs influence cell cycle progression by regulating these important components in HCC (**Figure 3**). For instance, Chen and coworkers find that exosome circ-deubiquitination (circ-DB) is upregulated in HCC patients with higher body fat ratios. The exosome circ-DB promotes HCC growth and reduces DNA damage *in vitro* and *in vivo* studies. Mechanistically, exosome circ-deubiquitination (exo-circ-DB) promotes HCC growth by activating USP7/cyclin A2 through sponging miR-34a (68). Has_circ_0078710 in HCC promotes cell proliferation by absorbing miR-31 and upregulating expression of HDAC2 and CDK2 (69). Circ-ZEB1.33 promotes the proliferation of HCC cells by regulating miR-200a-3p/CDK6 (70). Hsa_circ_0016788 accelerates HCC growth *via* regulation of miR-481/CDK4 (71). Moreover, hsa_circ_0091581 promotes proliferation of HCC cells by upregulating c-Myc by sponging miR-526b (72). circBACH1 interacts with HuR directly and promotes HuR accumulation in the cytoplasm to decrease p27 expression (73).

Instead, specific circRNAs as suppressor genes influence cell cycle progression in HCC. For example, circMTO1 suppresses proliferation of HCC cells by sponging miR-9 and increasing p21 expression (16). Furthermore, Chen and colleagues find that circLARP4 is downregulated in HCC and is associated with survival outcome for HCC patients. In vitro experiments show that circLARP4 inhibits cell proliferation, causes cell cycle arrest, and induces senescence in HCC. Mechanistically, circLARP4 inhibits HCC progression and induces cell cycle arrest by regulating miR-761/RUNX3/p53/p21 (74). Overexpressed hsa_circ_0079929 inhibits HCC cell proliferation and exerts cell cycle arrest by inhibiting cyclin B1 (75).

#### Angiogenesis Regulation by circRNAs in HCC

**Figure 4:**
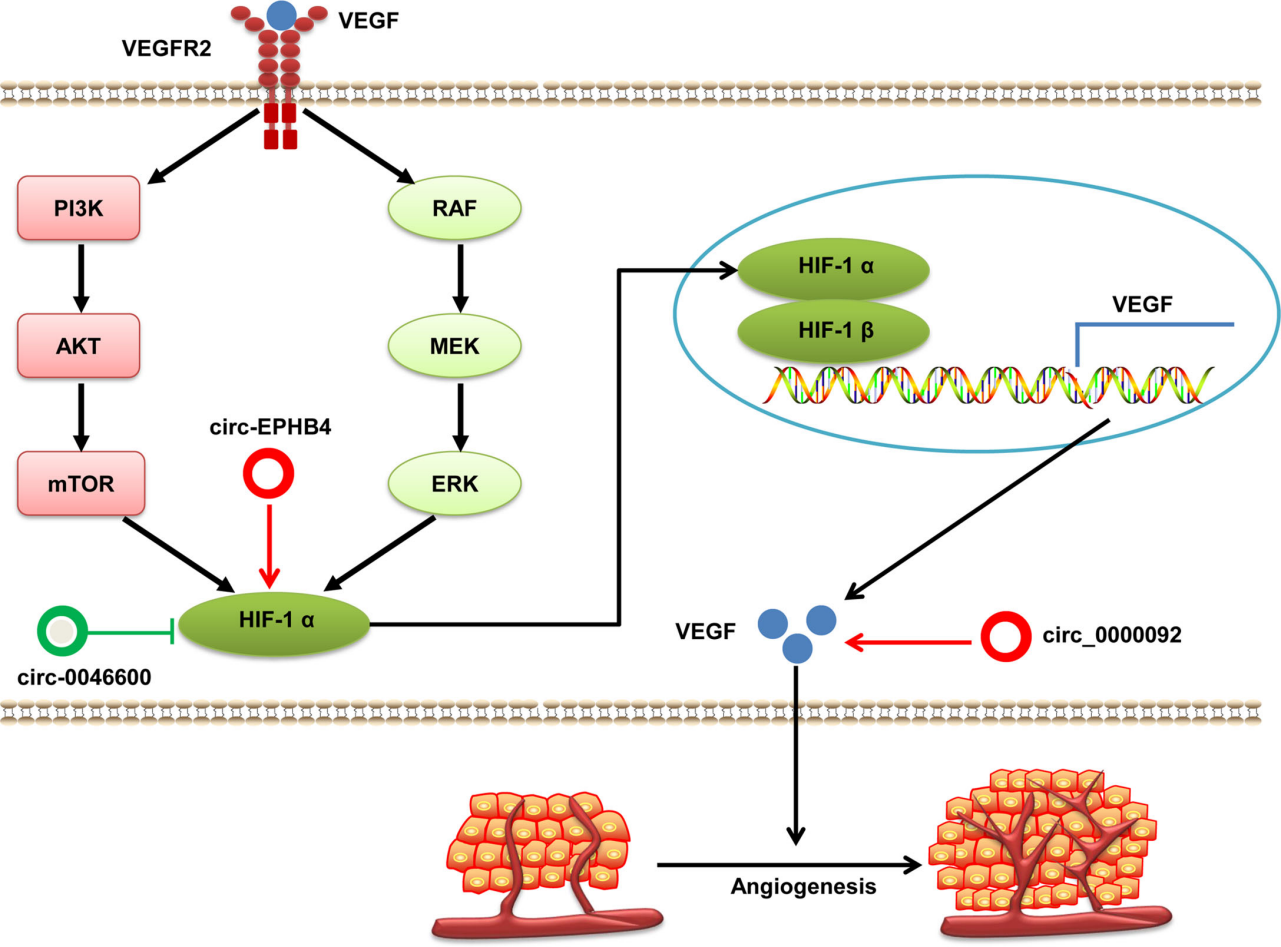
The regulatory role of circRNAs in HCC angiogenesis. VEGF protein binds with VEGFR 2 and activates the PI3K/AKT and MAPK/MEK signaling pathways, which induces HIF-1α expression. HIF-1α translocates into the nucleus and partners with HIF-1β to form a heterodimer that activates VEGF and promotes angiogenesis. Dysregulated circRNAs interfere with the expression of VEGF and HIF-1α in HCC. circRNAs with pro-angiogenic potential are denoted as red circles whereas anti-angiogenic circRNAs are denoted as green circles. HIF-1α, hypoxia-inducible factor 1α; VEGF, vascular endothelial growth factor; VEGFR2, vascular endothelial growth factor receptor 2.

Excessive abnormal angiogenesis is one of hallmarks of cancer. It is strongly associated with the growth, development, progression, and metastasis of tumor cells (76). This process is controlled by various angiogenic and antiangiogenic factors dominated by the tissue hypoxia-triggered overproduction of vascular endothelial growth factor (VEGF) (77). VEGF is the downstream gene of hypoxia-induced factor (HIF)-1 and the best studied angiogenic growth factor. VEGF induces mitogenesis and the migration of endothelial cells to form new blood vessels. Growth factors, produced by HIF-1 signaling, activate PI3K/Akt or mitogen-activated protein kinase/extracellular signal-regulated kinase (MAPK/MEK) signaling pathways. This action leads to increased expression of HIF-1 protein, which upregulates VEGF expression to promote cancer angiogenesis (77, 78). VEGF and HIF-1 can be regulated directly or indirectly by circRNAs in HCC (**Figure 4**). For example, Pu et al. finds that hsa_circ_0000092 expression is significantly increased in HCC tissues and cells lines. Depleted hsa_circ_0000092 suppresses HCC cells proliferation, migration, invasion and angiogenesis *in vitro* and *in vivo*. Mechanistic studies have revealed that hsa_circ_0000092 promotes angiogenesis in HCC by sponging miR-338-3p and upregulating expression of HN1, MMP9 and VEGF (79). Moreover, oncogenic circ-EPHB4 and tumor suppressor hsa-circ-0046600 modulate HIF-1a through different mechanisms in HCC (80, 81). Totally, studies on the roles of circRNAs associated with angiogenesis in HCC are limited, and additional research is needed to elucidate this issue.

#### Apoptosis Regulation by circRNAs in HCC

**Figure 5:**
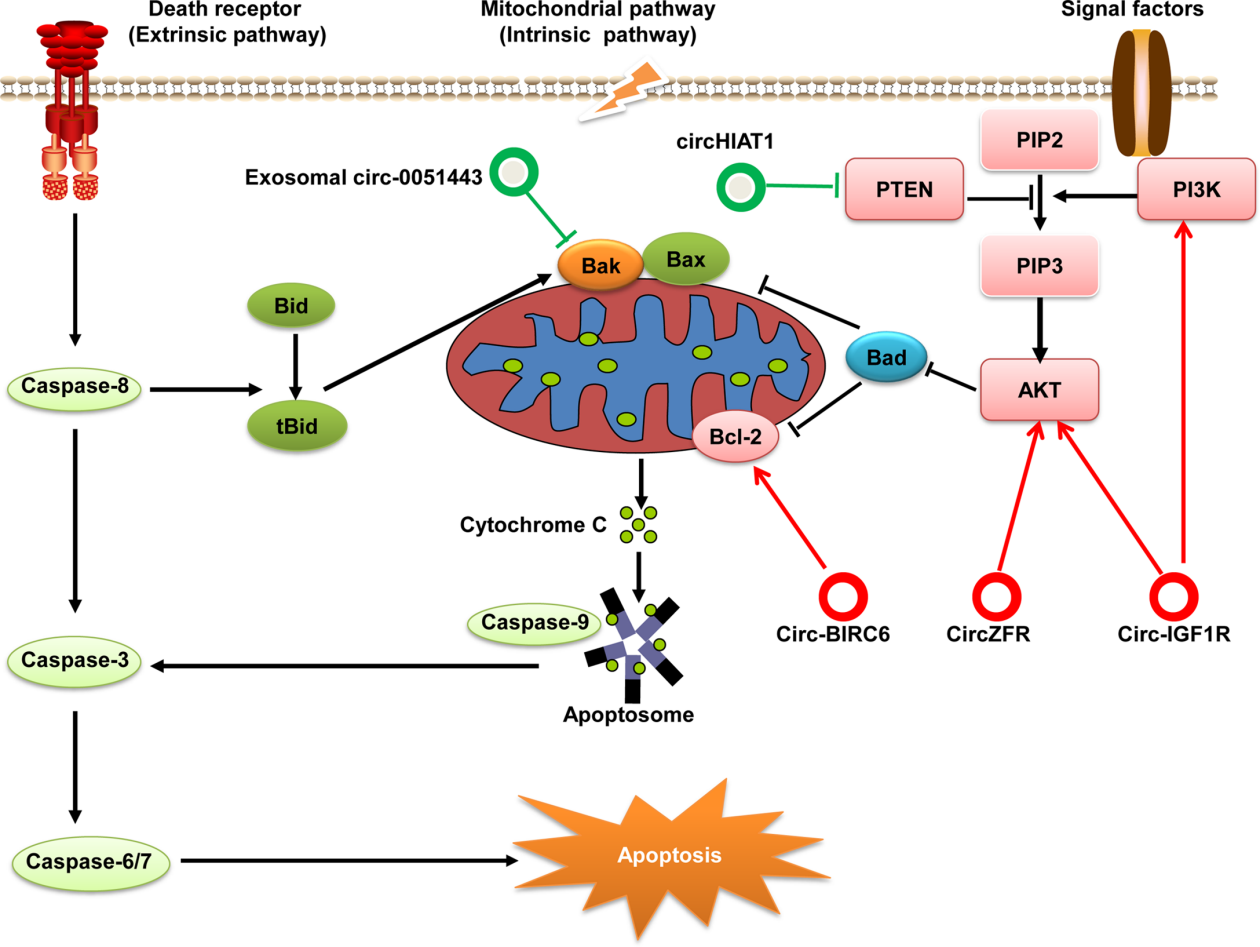
Regulatory role of circRNAs in the pathway for apoptosis of HCC. Apoptosis can be initiated by one of two pathways: extrinsic or intrinsic. Eventually, both pathways converge at the activation of executioner caspases that execute apoptosis. If PI3K is activated by cytokines or growth factors, it phosphorylates PIP2 to generate PIP3 and subsequently activates the PI3K/Akt signaling pathway. AKT signaling has a strong anti-apoptotic role through the phosphorylation and inhibition of key pro-apoptotic proteins (e.g., Bad). PTEN can dephosphorylate PIP3 to PIP2 and negatively regulates the PI3k/Akt pathway. These components involved in apoptosis can be regulated by circRNAs in HCC. circRNAs with anti-apoptotic effects are denoted as red circles whereas pro-apoptotic circRNAs are denoted as green circles.

One of hallmarks of cancer cells is the ability to evade apoptosis. Some studies have suggested that apoptosis is critically linked to the initiation and progression of HCC (82). Apoptosis (i.e., programmed cell death) can be initiated through one of two pathways: extrinsic and intrinsic. The extrinsic (“death receptor”) pathway is activated by the binding of extracellular ligands to death receptors, which leads to formation of a multi-protein complex called “death-inducing signaling complex”: this regulates activation of initiator caspase-8. The intrinsic (“mitochondrion-centered”) pathway of apoptosis is triggered upon loss of integrity of the mitochondrial outer membrane, resulting in the release of pro-apoptotic factors (e.g., cytochrome c) from the mitochondria into the cytosol (83). In the cytoplasm, cytochrome c forms a complex with apoptosis protease activating factor-1 (Apaf1) and caspase-9 by way of cascade amplification and continues to activate caspase-3, -6, and -7, eventually leading to apoptosis (84). Recent research has shown that circRNAs play an important part in apoptotic regulatory mechanisms in HCC by targeting the anti-apoptotic and pro-apoptotic components involved in apoptosis signaling pathways (**Figure 5**).

B-cell lymphoma (Bcl)-2 was the first gene shown to promote prolong cell survival rather than increase cell proliferation. Bcl-2 is an apoptosis-suppressor protein widely known for its significant roles in inhibiting apoptosis and promoting oncogenesis (85). circ-BIRC6 is significantly overexpressed in HCC tissue samples and associated with the overall survival of HCC patients. Knockdown of circ-BIRC6 expression promotes the apoptosis of HCC cells by modulating Bcl-2 expression *via* sponging miR-3918 (86). Furthermore, circ-0051443 expression is downregulated in HCC tissues and plasma; and exosomal circ-0051443 suppresses the biological behaviors of HCC cells by promoting apoptosis *via* sponging miR-331-3p and regulation of Bak 1 (87).

Moreover, some signaling pathways also participate in apoptosis regulation *via* anti-apoptotic or pro-apoptotic proteins. The PI3K/Akt signaling pathway plays an important part in regulating many cellular and biological functions, including apoptosis (88). While the tumor suppressor phosphatase and tensin homolog (PTEN) has been identified as a negative regulator of the PI3K/Akt signaling pathway (89). PI3K is activated by receptor tyrosine kinases, which leads to Akt activation. Recent data have shown that specific circRNAs exert regulatory roles on the PTEN/PI3K/Akt signaling pathway. For example, circ-IGF1R expression is significantly upregulated in HCC tissues and the high expression levels of circ-IGF1R is associated with tumor size. Knocking down circ-IGF1R induces cell apoptosis and cell cycle arrest *in vitro*. Mechanistic studies have revealed that circ-IGF1R exerts anti-apoptotic effects by activating the PI3K/Akt pathway (90). Similarly, another study performed by Yang et al. shows that circZFR is significantly increased in HCC tissues and cells. Silencing circZFR inhibits HCC cell proliferation, migration and invasion, and induced apoptosis of HCC cells. Mechanistically, circZFR inhibits apoptosis and promotes cell proliferation through regulating the miR-511/Akt1 axis (91). However, circHIAT1 expression is significantly downregulated in HCC and inhibits apoptosis by targeting the miR-3171/PTEN axis (92).

#### circRNAs Are Associated With Regulation of EMT in HCC

Epithelial–mesenchymal transition (EMT) plays a critical part in the invasion and metastasis of cancer cells. EMT is characterized by a loss of epithelial markers (e.g., E-cadherin) and increased expression of mesenchymal markers (e.g., N-cadherin, Vimentin, and Fibronectin) driven initially by several EMT transcriptional factors, including snail, zeb, twist and slug families (93–95). Moreover, certain signaling pathways (e.g., Wnt/β-catenin and nuclear factor-kappa B (NF-κB), oncogenes, and tumor suppressors also participate in regulating EMT.

**Figure 6:**
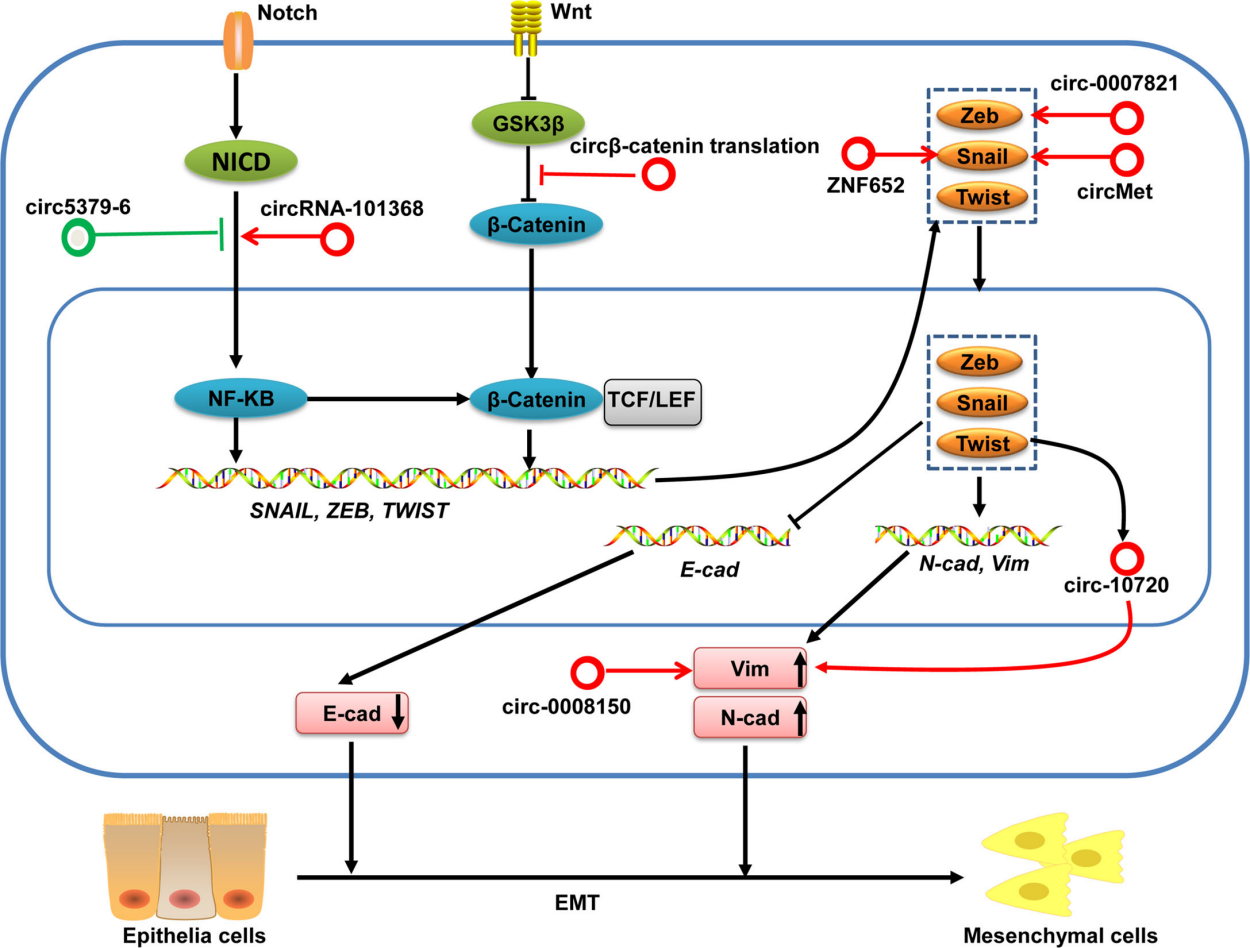
circRNAs in the EMT regulation in HCC. Activation of Wnt/β-catenin and Notch/NF-KB pathways can induce various EMT-transcription factors. These EMT-transcription factors subsequently lead to repression of epithelial markers (e.g., E-cadherin) expression and increased expression of mesenchymal markers (e.g., N-cadherin and Vimentin), which finally facilitate the EMT. These EMT-associated transcription factors and signaling pathways can be regulated by circRNAs in HCC. circRNAs with pro-EMT effects are denoted as red circles whereas circRNAs with anti-EMT effects are denoted as green circles. Vim, Vimentin; N-cad, N-cadherin; E-cad, E-cadherin.

Several circRNAs have been shown to influence the invasion and metastasis of HCC by regulating EMT-transcription factors, Wnt/β-catenin and NF-κB signaling pathways in HCC (**Figure 6**). For example, circMET is overexpressed in HCC tumors and the increased expression of circMET is associated with survival and recurrence for HCC patients. In vitro experiments find that circMET overexpression promoted HCC development by inducing EMT and enhancing the immunosuppressive tumor microenvironment. Mechanistically, circMET promotes HCC development by inducing EMT *via* miR-30-5p/Snail (96). circ-ZNF652 increases snail expression by sponging miR-203 and miR-502-5p, which results in promotion of metastasis of HCC (6). Han et al. demonstrates that circ-0008150 upregulates vimentin expression by sponging miR-615-5p, whereas circ-0007821 inhibits E-cadherin expression by absorbing miR-381-3p-targeted zeb1 (97). Furthermore, Meng and colleagues unveiled a mechanism by which the EMT-transcription factor twist1 regulated vimentin expression through circ-10720 to promote EMT in HCC (98). In addition, translation of circβ-catenin (a 370 amino-acid β-catenin isoform) promotes metastasis of HCC by activating the Wnt/β-catenin pathway (19). Moreover, circZFR has an oncogenic role in HCC through regulating the miR-3619-5p/CTNNB1 axis and activating the Wnt/β-catenin signaling pathway (99). circRNA-101368 modulates migration and metastasis of HCC through miR-200a and downstream HMGB1/RAGE/NF-κB signaling (100). Conversely, circ5379-6 inhibits the metastasis of HCC *via* targeting of the NF-κB signaling pathway (101).

#### Regulation of Metabolism in HCC by circRNAs

**Figure 7:**
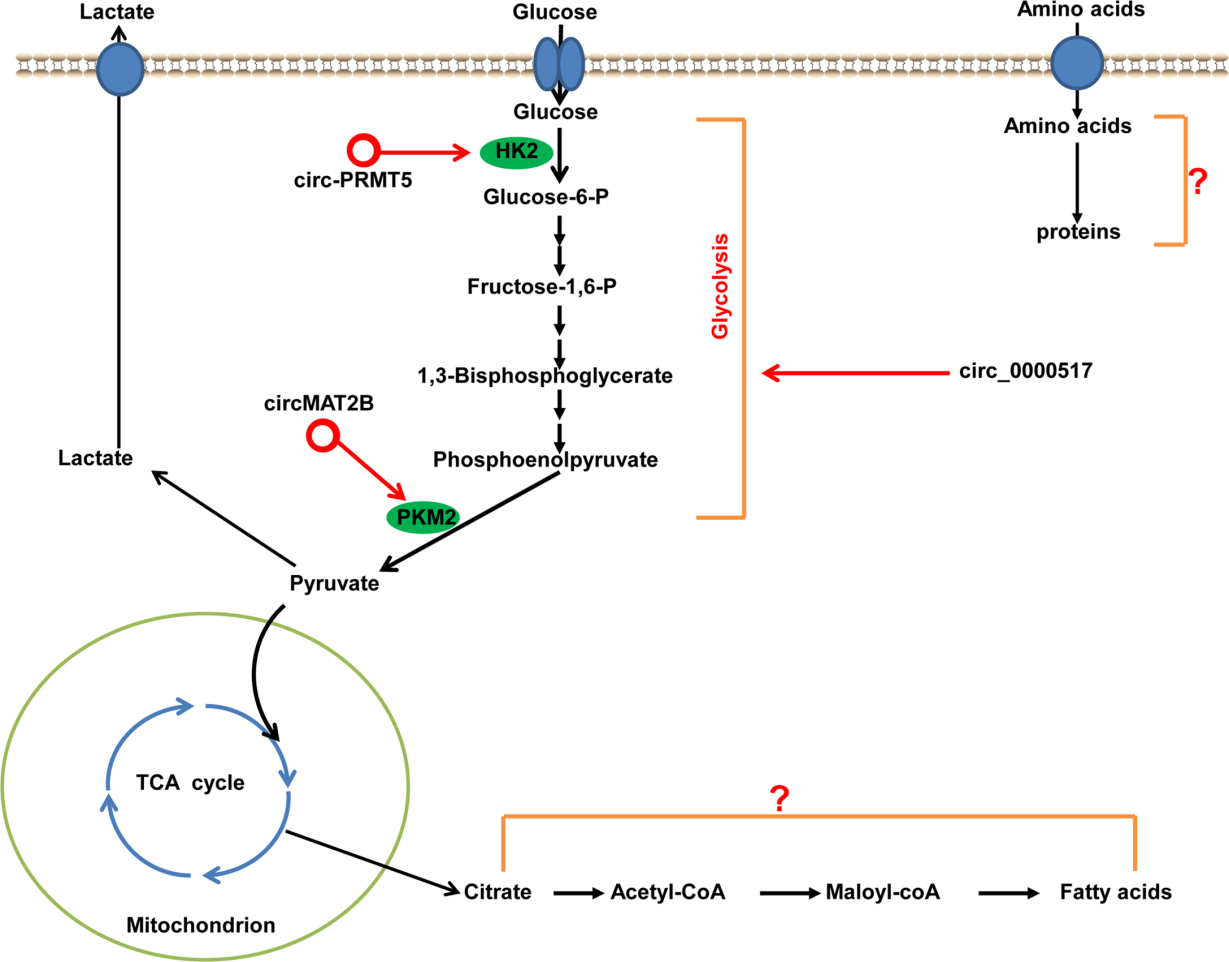
Metabolic regulation by circRNAs in HCC. HK2 and PKM2 are important rate-limiting enzymes in the first and last steps of glycolysis, which can be regulated by circRNAs in HCC. circRNAs with pro-glycolysis effects are denoted as red circles. The roles and mechanisms of circRNAs in the regulating of metabolism of lipids and amino acids in HCC have not been discovered. PMK2, pyruvate kinase 2; HK2, hexokinase 2.

Dysregulated metabolism is a common feature of cancer cells, and is considered one of the hallmarks of cancer (102). To meet the energy demands for rapid growth, proliferation, invasion, and metastasis, tumor cells “reprogram” their energy metabolism, mainly the metabolism of glucose, lipids, and amino acids. The most characteristic metabolic alteration in tumors is reprogramming of glucose metabolism, which is referred to as glucose metabolic change from oxidative metabolism to aerobic glycolysis (“Warburg effect”). Glycolysis in tumor cells is controlled precisely by a series of oncogenes, tumor-suppressor genes, signaling pathways, and genes encoding key enzymes related to glycolysis (e.g., PKM2 and HK2), all of which may be regulated by circRNAs to facilitate HCC development (**Figure 7**). For example, Li et al. finds that circMAT2B expression is significantly upregulated in HCC tissues and cell lines. The increased expression of circMAT2B is strongly associated with glycolysis in HCC patients. circMAT2B promotes glycolysis and glycolysis-related cell proliferation, migration, and invasion *in vitro* under hypoxia. *In vivo* studies show that increased expression circMAT2B overexpression increases HCC glucose utilization, tumor growth, and metastasis. Mechanistically, circMAT2B promotes HCC progression by increased glycolysis by activating circMAT2B/miR-338-3p/PKM2 under hypoxia (103). Ding and coworkers find that circ-PRMT5 is significantly increased in HCC tissues and cells. Loss-of-functional experiments show that the silencing of circ-PRMT5 inhibits HCC cells proliferation, migration, glycolysis *in vitro* and tumor growth *in vivo*. Mechanistically, circ-PRMT5 promotes HCC proliferation, migration and glycolysis by miR-188-5p sponging to regulate HK2 (104). Furthermore, circ-0000517 promotes HCC progression and glycolysis *via* miR-326/IGF1R (105). In total, circRNAs are novel ncRNAs, and only a few studies have reported on the altered metabolic reprogramming regulated by circRNAs in HCC. Theoretically, circRNAs can not only regulate the programming of glycolysis in HCC, but also modulate the programming of lipids and amino acids, though this have not been reported yet.

### circRNAs Regulate the Stemness of Cancer Cells in HCC

Cancer stem cells (CSCs) are a small and rare subpopulation of cells within tumors with self-renewal, differentiation, and tumor-initiating capabilities (106, 107). CSCs are considered to be the “seeds” of tumors. CSCs have a significant role in tumor relapse, metastasis, resistance against therapy, and are targets for cancer treatment (108). Several studies have demonstrated that specific circRNAs can influence CSCs in HCC by different mechanisms. For example, Zhu et al. performs sequencing to identify the expression patterns of circRNAs and finds that circZKSCAN1 expression is decreased in HCC tissues. By using 112 pairs of HCC tissues, they identify a negative correlation between epithelial cell adhesion molecule (EpCAM) mRNA and circZKSCAN1 expression. circZKSCAN1 inhibits malignant behavior of HCC by modulating cell stemness. Mechanistically, circZKSCAN1 to suppress cell stemness in HCC by regulating the function of the RBP fragile X mental retardation protein (FMRP) and blocking the binding between FMRP and β-catenin-binding protein-cell cycle and apoptosis regulator 1, thereby subsequently reducing the activity of the Wnt/β-catenin pathway (109). Moreover, Chen and coworkers found a novel mechanism by which circ-MALAT1 acted as a “brake” in ribosomes to prevent translation of PAX5 mRNA and thus promoted CSCs self-renewal in HCC by forming a ternary complex between ribosomes, mRNA and circ-MALAT1. Simultaneously, circ-MALAT1 could promote CSCs self-renewal by absorbing miR-6887-3p and enhancing the JAK2/STAT3 pathway. Therefore, they uncovered a novel dual-faceted regulatory mechanism by a circRNA for promoting CSCs self-renewal in HCC (110).

### circRNAs and Immunotherapy in HCC

Immunotherapy with checkpoint blockade has become an important weapon in fighting cancer (111). Immunotherapy based on blockade of the programmed death-1/programmed death ligand-1 (PD-1/PD-L1) checkpoint represents a common strategy for several types of tumors, including HCC (112, 113). However, the overall response rates are unsatisfactory and adverse events have been observed, which suggests an urgent need to understand the basic biology of cancer immunosuppression (114). Emerging data have shown that certain circRNAs can induce immunosuppression and resistance to anti-PD1 therapy in HCC. For example, circMET is an oncogenic circRNA that induces immunosuppression in HCC through the Snail/DPP4/CXCL10 axis. Importantly, the DPP4 inhibitor sitagliptin can increase infiltration of CD8^+^T cells in HCC tissues from patients sufferering from diabetes mellitus. Hence, a DPP4 inhibitor could significantly enhance the efficacy of immunotherapy based on PD1 blockade in a subgroup of HCC patients (96).

Furthermore, infiltration by and the function of natural killer (NK) cells improves survival in patients with cancer. This phenomenon provides novel opportunities to target NK cell function to increase immunotherapy response rates in various malignant tumors, including HCC (115). Some research has demonstrated NK-cell dysfunction to occur in HCC. However, the mechanisms underlying NK cell dysfunction in HCC are not clearly understood. Zhang and colleagues found that circUHRF1 promoted the progression and immunosuppression of HCC in an exosome- and NK cell-dependent manner (116). Mechanistically, circUHRF1 inhibited NK cell function by sponging miR-449c-5p and promoting TIM-3 expression. Importantly, circUHRF1 could drive resistance to anti-PD1 immunotherapy. Thus, targeting circUHRF1 might be a promising strategy for improving the therapeutic efficacy of anti-PD1 immunotherapy in HCC. In total, circRNAs may become a novel research direction for tumor immunotherapy.

## Potential Clinical Applications of CIRCRNAs in HCC

The potential clinical applications of circRNAs in HCC have garnered increasing attention. circRNAs are abundant, stable, evolutionally conserved, and disease-associated expression signatures. These unique characteristics could enable circRNAs to be employed in the diagnosis and prognosis of cancer (5). Furthermore, circRNAs are involved in expression regulation and tumor progression by distinct mechanisms. Hence, circRNAs could be therapeutic targets and players in mediating therapy resistance for HCC (117, 118). Representative circRNAs are shown in **Table 1**.

**Table 1:** Representative dysregulated circRNAs and potential clinical applications in HCC.

CircRNAs	Expression	Mechanism	Target genes and signaling pathway	Clinical application	References
circRHOT1	↑	regulation gene transcription	TIP60-dependent NR2F6 expression	Prognosis	(119)
SCD-circRNA 2	↑	regulated by RBM3	SCD-circRNA2/ERK pathway	Prognosis	(120)
circRNA_104348	↑	miRNA sponge	miR-187-3p/RTKN2	Prognosis	(121)
circCAMSAP1	↑	miRNA sponge	miR-1294/GRAMD1A pathway	Prognosis and therapeutic target	(122)
circTRIM33–12	↓	miRNA sponge	miR-191/TET1	Prognosis and therapeutic target	(123)
circDLC1	↓	Interaction RBPs	circDLC1-HuR-MMP1 axis	Prognosis and therapeutic target	(124)
circ_0091570	↓	miRNA sponge	miR-1307/ISM1	Diagnosis and prognosis	(125)
circ_0014717	↓	miRNA sponge	miR-668-3p/BTG2	Prognosis	(126)
circPABPC1	↓	Protein scaffold	circPABPC1 linked ITGB1 to proteasome for degradation	Prognosis and therapeutic target	(127)
exosomal circAKT3	↑	−	−	Prognosis	(128)
exosomal circ-0004277	↑	−	−	Diagnosis and therapeutic target	(129)
circFBXO11	↑	miRNA sponge	miR-605/FOXO3/ABCB1 axis	Diagnosis, therapeutic target and chemotherapy resistance	(130)
circ_0003418	↓	−	Wnt/β-catenin pathway	Diagnosis, therapeutic target and chemotherapy resistance	(131)
circRNA_101505	↓	miRNA sponge	miR-103/ONR1	Therapeutic target and chemotherapy resistance	(132)
cZNF292	↑	Bind to proteins	SOX9 nuclear translocation and Wnt/β-catenin pathway	Enhanced radiosensitivity	(133)
circRNA-SORE	↑	miRNA sponge	Sponging miR-103a-2-5p/miR-660-3p and activating Wnt/β-catenin pathway	Chemotherapy resistance	(134)
circRNA-SORE	↑	Bind to proteins	Bound and stabilized YBX1	Chemotherapy resistance	(135)
circASAP1	↑	miRNA sponge	miR-326/miR-532-5p-MAPK1/CSF-1 signaling	Prognosis and therapeutic target	(136)
circTMEM45A	↑	miRNA sponge	miR-665/IGF2 axis	Diagnostic and therapeutic target	(137)
Circ-CDYL	↑	miRNA sponge	miR-892a-HDGF/PI3K/AKT and miR-3283p/HIF1AN/NOTCH	Early diagnosis and therapeutic target	(138)
cSMARCA5	↓	miRNA sponge	miR-17-3p/miR-181b-5p-TIMP3 pathway	Prognosis and therapeutic target	(17)
CircMTO1	↓	miRNA sponge	miR-9/p21 pathway	Prognosis and therapeutic target	(16)

### circRNAs as Diagnostic and Prognostic Biomarkers for HCC

According to identification by RNA-sequencing/microarray and validation by real-time reverse transcription-quantitative polymerase chain reaction (RT-qPCR), some circRNAs with upregulated/downregulated expression may serve as potential biomarkers in the diagnosis and prognosis for HCC. For example, circRHOT1 expression is substantially upregulated in HCC tissues, and high tissue circRHOT1 levels are associated with the clinical stage and a worse prognosis. Therefore, circRHOT1 may serve as a potential biomarker for HCC prognosis (119). Furthermore, increased expression of SCD-circRNA 2 and has_circ_104348 can predict a poor prognosis in HCC patients and serve as potential prognostic biomarkers for HCC (120, 121). Recently, Luo and colleagues found that circCAMSAP1 expression was significantly increased in HCC tissues. This increased expression of circCAMSAP1 promoted the biological functions of HCC *in vitro* and *in vivo*. Mechanistically, circCAMSAP1 promoted HCC progression through the miR-1294/GRAMD1A pathway. Therefore, circCAMSAP1 might be a potential prognostic and therapeutic target for HCC (122).

Besides upregulated expression of circRNAs, downregulated expression of circRNAs can also serve as a potential biomarker for HCC. For example, decreased expression of circTRIM33-12 and circDLC1 can predict a poor clinical outcome, indicating their prognostic value for HCC (123, 124). Furthermore, downregulated expression of hsa_circ_0091570 could serve as a potential diagnostic and prognostic marker for HCC (125). Recently, Ma and colleagues found that circ_0014717 expression was significantly decreased in HCC, and the decreased expression of circ_0014717 was associated with the overall survival and the time to tumor recurrence. Overexpression of circ_0014717 inhibited the growth, migration, and invasion of HCC cells *in vitro* and *in vivo*. Mechanistically, circ_0014717 inhibited HCC tumorigenesis by regulating the miR-668-3p/BTG2 axis. Therefore, circ_0014717 might be a potential prognostic biomarker for HCC (126). Shi and coworkers found circPABPC1 expression to be significantly decreased in HCC tissues, and that decreased expression of circPABPC1 in HCC was strongly correlated with shortened overall survival and disease-free survival. As a tumor suppressor in HCC, circPABPC1 physically links ITGB1 (β1 integrin) to proteasomes for degradation in a ubiquitin-independent manner, thereby inhibiting the adhesion and migration of cells. Therefore, circPABPC1 might be a potential prognostic and therapeutic target for HCC (127).

In addition, exosomal circRNAs are detected readily and also serve as potential biomarkers for HCC. For example, upregulated expression of exosomal circAKT3 in HCC might act as a prognostic marker for HCC after surgical treatment (128). Furthermore, exosomal circ-0004277 expression is significantly increased in the plasma of HCC patients. The increased expression of exosomal circ-0004277 in HCC presents good diagnostic value because its area under the curve (AUC), sensitivity, and specificity are 0.816, 58.3% and 96.7%, respectively. Those results demonstrated that exosomal circ-0004277 might be a useful diagnostic biomarker and therapeutic target for HCC patients (129).

### circRNAs as Players in Mediating Therapy Resistance to HCC

Therapy resistance is an important factor for the recurrence and metastasis of HCC, and one of the biggest obstacles to tumor treatment (139). Recently, several studies have found that some dysregulated circRNAs are involved in the resistance of HCC to chemotherapy and radiotherapy. For example, circRBXO11 promotes the tumor progression and oxaliplatin resistance of HCC cells by sponging miR-605, targeting FOXO3, and activating ABCB1 (130). Moreover, circ_0003418 inhibits tumorigenesis and cisplatin chemoresistance *via* the Wnt/β-catenin pathway in HCC (131). Furthermore, circRNA_101505 sensitizes HCC cells to cisplatin by absorbing miR-103 and upregulating expression of NOR1 (132). Also, cZNF292 enhances the radiosensitivity of hypoxic HCC cells by increasing SOX9 nuclear translocation and reducing Wnt/β-catenin pathway (133). Besides, Wu et al. found circRNAs profile of HCC might be used as potential biomarkers for sorafenib-resistant HCC patients (140). Xu et al. recently found that increased expression of circRNA-SORE maintained sorafenib resistance in HCC mainly through two mechanisms: (i) sponging miR-103a-2-5p/miR-660-3p and activating the Wnt/β-catenin pathway; in particular, N6-methyladenosine-modified circRNA-SORE increased circRNA-SORE expression by increasing RNA stability (134); (ii) circRNA-SORE bound and stabilized YBX1 by preventing PRP19-mediated degradation of YBX1; in addition, circRNA-SORE (which was transported by exosomes) could transmit sorafenib resistance among HCC cells (135).

### circRNAs as Potential Therapeutic Targets for HCC

Specific circRNAs with upregulated expression are intimately involved with HCC progression, so circRNAs could be therapeutic targets for HCC. For example, circASAP1 is overexpressed in HCC cells and in patients who experience HCC metastasis or recurrence. circASAP1 promotes the proliferation, colony formation, migration, invasion, tumor growth and pulmonary metastasis of HCC cells *in vitro* and *in vivo*. Mechanistic studies have revealed that circASAP1 promotes HCC metastasis *via* miR-326/miR-532-5p-MAPK1/CSF-1 signaling. Therefore, circASAP1 could function as a novel therapeutic target for HCC (136). Furthermore, increased circTMEM45A expression in serum exosomes from HCC patients may act as a novel diagnostic and therapeutic target for HCC patients (137). Moreover, a regulatory network of circ-CDYL-centric ncRNAs combined to HDGF and HIF1AN may function as promising biomarkers and targets for the early diagnosis and treatment of HCC (138). Therefore, upregulated expression of these circRNAs in HCC may have oncogenic roles, and silencing expression of these circRNAs have opposite effects in HCC. Small-interfering RNAs that are designed to target the backsplicing junction sites of oncogenic circRNAs can inhibit the development of HCC and exhibit an antitumor effect (141, 142).

Some circRNAs with downregulated expression act as tumor suppressors and inhibit HCC development, also making them potential therapeutic targets. For example, cSMARCA5 expression is significantly downregulated in HCC tissues, is correlated with growth and metastasis, and could serve as an independent prognostic marker for HCC patients after hepatectomy. The *in vivo* and *in vitro* intervention of cSMARCA5 indicates that cSMARCA5 inhibits growth and metastasis in HCC. Therefore, cSMARCA5 could be a prognostic and therapeutic target for HCC (17). Han et al. found that downregulated expression of circMTO1 was associated with a poor prognosis for HCC patients. Intratumoral knockdown of circMTO1 expression enhanced HCC growth *in vivo*, indicating its potential in HCC-targeted therapy. circMTO1 may act as a novel prognostic biomarker and therapeutic target for HCC patients (16). Zhang et al. found that downregulated expression of circDLC1 in HCC was closely related to the prognosis for HCC patients. Overexpression of circDLC1 inhibited the proliferation and metastasis of HCC cells *in vitro* and *in vivo*. circDLC1 may act as a prognostic biomarker and therapeutic target for HCC patients (124). For these circRNAs with downregulated expression, the induction of their overexpression in HCC cells or tissues *via* transfection could yield significant anticancer effects.

In total, such dyeregulated expression of circRNAs in HCC can act as tumor activators or tumor suppressors and be a therapeutic targets for HCC intervention.

## Perspectives, Challenges and Conclusions

Many dysregulated circRNAs have been discovered and identified in HCC tissue. Analyses of the expression profile of circRNAs using RNA sequencing or microarray technology has shown that some dysregulated circRNAs have important roles in HCC. Specific circRNAs are correlated with HCC carcinogenesis and progression, including the proliferation, angiogenesis, apoptosis, invasion and migration, metabolism, and evading destruction by the immune system. Furthermore, clinicians and researchers are extremely eager to find sensitive and reliable circRNAs to improve the early diagnosis, assessment of treatment efficacy, and prognosis prediction of HCC by analyzing circRNAs expression profile combined with real-time RT-qPCR.

Though much progress has been made on circRNAs, the study of circRNAs in HCC is in its infancy. Compared with other ncRNAs, such as miRNAs and IncRNAs, only a small part of functional circRNAs have been identified in HCC. The study of circRNAs also faces six major challenges that need further exploration.

First, a standardized nomenclature system is needed to reduce inefficient work and confusion among circRNAs researchers employing different circRNAs databases. We suggest that the naming of circRNAs should contain the name of the host gene along with the term “circ” in the prefix (143–145). Second, knowledge on the mechanism of biogenesis and circularization of circRNAs is limited. In particular, the degradation and metabolic processing of circRNAs in cells is not known. Third, more sensitive detection methods should be developed to improve detection of circRNAs from specimens with low RNA quality (146). Fourth, the overlap in contents between different circRNAs databases is limited. Therefore, we suggest running multiple circRNAs-associated databases or combining different databases to discover novel circRNAs and predict the mechanisms of circRNAs (147). Fifth, most studies of circRNAs for HCC have been conducted in relatively small sample sizes with a lack of standard protocols for sample processing. Future studies with large-scale clinical samples and standardization of sample processing investigating the differential expression of circRNAs in patients with HCC are warranted. Finally, circRNAs-targeted treatment of HCC is a novel research direction. Therefore, additional studies are needed on how to transport artificial circRNAs efficiently and accurately to the action site without immune rejection (148). The answers to these questions will contribute to understanding of the roles of circRNAs in cancer, including HCC. We also believe these problems will be solved gradually in future studies.

Here, we wish to emphasize the importance of exploring other mechanisms of circRNAs in addition to miRNA sponges, such as interaction with RBPs and protein translation. Discovery of a novel regulatory mechanism of circRNAs in HCC (e.g., a mRNA brake) (110) will hopefully open a new path for circRNAs roles in regulating tumorigenesis and progression. In particular, we highlight that circRNAs in the regulation of immune escape, stemness of cancer cells, metabolism and therapy resistance in HCC may become important directions in future studies in HCC.

Taken together, circRNAs are produced mainly by backsplicing and categorized into four types: ecircRNAs, EIciRNAs, ciRNAs and tricRNAs. Furthermore, circRNAs can have diverse roles, including sponging miRNAs, encoding peptides, binding to RBPs, promoting gene transcription, and regulating alternative splicing. The functions of circRNAs in HCC have become “hot topics” of research, and specific circRNAs have been demonstrated as having crucial roles in the carcinogenesis and progression of HCC. Hence, circRNAs could have clinical applications in the diagnosis, prognosis and therapy for HCC. Moreover, great advancements with regard to the functions of circRNAs in tumor hallmarks, stemness of cancer cells, therapy resistance and immunotherapy have been revealed. Although many challenges and questions remain unanswered, we believe, accompanied by the development of research strategies, sequencing technologies and the use of online databases, future studies in the biogenesis, functions, and clinical relevance of circRNAs in HCC will improve understanding of the role of circRNAs in cancer biology.

## Author Contributions

YZ collected the literature, and conceived and wrote the manuscript. YW wrote and reviewed the manuscript critically. All authors contributed to the article and approved the submitted version.

## Funding

This research was funded by National Natural Science Foundation of China (81702896); Science and Technology Plan Projects of Jilin Province (20200201539JC); Department of Education of Jilin Province (JJKH20180198KJ); China Scholarship Council (201706175044 and 201606175202); and Jilin University Project (2019YX396).

## Conflict of Interest

The authors declare that the research was conducted in the absence of any commercial or financial relationships that could be construed as a potential conflict of interest.

## Glossary

**Table:**

HCC	Hepatocellular carcinoma
ncRNAs	non-coding RNAs
pre-mRNAs	precursor messenger RNAs
ecircRNAs	exonic circRNA
EIciRNAs	retained-intron or exon-intron circRNAs ciRNAs intronic circRNAs
tricRNAs	tRNA intronic circRNAs
miRNAs	microRNAs
RBPs	RNA-binding proteins
QKI	quaking
MBL	muscleblind proteins
ADAR1	adenosine deaminases acting on RNA 1
SMN	Survival of Motor Neuron
CDR1	cerebellum degeneration-related protein antisense
PABPN1	Poly(A)-binding protein nuclear 1
YAP	Yes-associated protein
PI3K/Akt	phosphoinositide 3-kinase/protein kinase B
U1 snRNP	U1 small nuclear ribonucleoprotein
snRNA	small nuclear RNA
Pol II	RNA polymerase II
CKIs	CDK inhibitor proteins
INK4	Inhibitors of CDK4
Cip/Kip	CDK interacting protein/Kinase inhibitory protein
USP7	ubiquitin-specific protease 7
HDAC2	histone deacetylase 2
RUNX3	Runt-related transcription factor 3
VEGF	vascular endothelial growth factor
MAPK/MEK	mitogen-activated protein kinase/extracellular signal-regulated kinase
HIF-1	hypoxia-induced factor-1
HN1	haematopoietic-and neurologic-expressed sequence 1
MMP9	matrix metalloproteinase 9
Apaf1	apoptosis protease activating factor-1
Bcl-2	B-cell lymphoma-2
PTEN	phosphatase and tensin homolog
EMT	Epithelial-mesenchymal transition
NF-κB	nuclear factor-kappa B
Bak1	BCL2 antagonist/killer 1
HMGB1	high-mobility group box 1 protein
RAGE	receptor for advanced glycation end products
Wnt	wingless type
JAK2/STAT3	Janus kinase 2/signal transducer and activator of transcription 3
PKM2	pyruvate kinase 2
HK2	Hexokinase 2
CSCs	Cancer stem cells
FMRP	fragile X mental retardation protein
PAX5	Paired box 5
DPP4	dipeptidyl peptidase 4
TIM-3	T cell immunoglobulin and mucin domain 3
GRAMD1A	GRAM domain-containing protein 1A
BTG2	B-cell translocation gene 2
ITGB1	β1 integrin
ABCB1	multidrug resistance protein 1
FOXO3	Forkhead Box O3
NOR1	oxidored-nitro domain-containing protein 1
SOX9	sex determining region Y-box 9
YBX1	Y box-binding protein 1
PRP19	Processing Factor 19
CSF-1	colony stimulating factor CSF-1
HDGF	hepatoma-derived growth factor
HIF1AN	hypoxia-inducible factor asparagine hydroxylase
HCC	H precursor messenger RNAs
ecircRNAs	exonic circRNA
EIciRNAs	retained-intron or exon-intron circRNAs ciRNAs intronic circRNAs
tricRNAs	tRNA intronic circRNAs
miRNAs	microRNAs
RBPs	RNA-binding proteins
QKI	quaking
MBL	muscleblind proteins
ADAR1	adenosine deaminases acting on RNA 1
SMN	Survival of Motor Neuron
CDR1	cerebellum degeneration-related protein antisense
PABPN1	Poly(A)-binding protein nuclear 1
YAP	Yes-associated protein
PI3K/Akt	phosphoinositide 3-kinase/protein kinase B
U1 snRNP	U1 small nuclear ribonucleoprotein
snRNA	small nuclear RNA
Pol II	RNA polymerase II
CKIs	CDK inhibitor proteins
INK4	Inhibitors of CDK4
Cip/Kip	CDK interacting protein/Kinase inhibitory protein
USP7	ubiquitin-specific protease 7
HDAC2	histone deacetylase 2
RUNX3	Runt-related transcription factor 3
VEGF	vascular endothelial growth factor
MAPK/MEK	mitogen-activated protein kinase/extracellular signal-regulated kinase
HIF-1	hypoxia-induced factor-1
HN1	haematopoietic-and neurologic-expressed sequence 1
MMP9	matrix metalloproteinase 9
Apaf1	apoptosis protease activating factor-1
Bcl-2	B-cell lymphoma-2
PTEN	phosphatase and tensin homolog
EMT	Epithelial-mesenchymal transition
NF-κB	nuclear factor-kappa B
Bak1	BCL2 antagonist/killer 1
HMGB1	high-mobility group box 1 protein
RAGE	receptor for advanced glycation end products
Wnt	wingless type
JAK2/STAT3	Janus kinase 2/signal transducer and activator of transcription 3
PKM2	pyruvate kinase 2
HK2	Hexokinase 2
CSCs	Cancer stem cells
FMRP	fragile X mental retardation protein
PAX5	Paired box 5
DPP4	dipeptidyl peptidase 4
TIM-3	T cell immunoglobulin and mucin domain 3
GRAMD1A	GRAM domain-containing protein 1A
BTG2	B-cell translocation gene 2
ITGB1	β1 integrin
ABCB1	multidrug resistance protein 1
FOXO3	Forkhead Box O3
NOR1	oxidored-nitro domain-containing protein 1
SOX9	sex determining region Y-box 9
YBX1	Y box-binding protein 1
PRP19	Processing Factor 19
CSF-1	colony stimulating factor CSF-1
HDGF	hepatoma-derived growth factor
HIF1AN	hypoxia-inducible factor asparagine hydroxylase
